# A Systematic Review on Clinimetric Properties of Play Instruments for Occupational Therapy Practice

**DOI:** 10.1155/2020/2490519

**Published:** 2020-07-17

**Authors:** Muhammad Hibatullah Romli, Farahiyah Wan Yunus

**Affiliations:** ^1^Department of Nursing and Rehabilitation, Faculty of Medicine and Health Sciences, Universiti Putra Malaysia, 43400 Serdang, Selangor, Malaysia; ^2^Malaysian Research Institute on Ageing (MyAgeing), Universiti Putra Malaysia, 43400 Serdang, Selangor, Malaysia; ^3^Centre for Rehabilitation and Special Needs Studies, Occupational Therapy Programme, Faculty of Health Sciences, Universiti Kebangsaan Malaysia, 50300 Kuala Lumpur, Malaysia

## Abstract

Play is considered the main occupation for children. Pediatric occupational therapists utilize play either for evaluation or intervention purpose. However, play is not properly measured by occupational therapists, and the use of play instrument is limited. This systematic review was aimed at identifying play instruments relevant to occupational therapy practice and its clinimetric properties. A systematic search was conducted on six databases (Academic Search Complete, CINAHL, MEDLINE, Psychology and Behavioral Science Collection, Scopus, and ASEAN Citation Index) in January 2020. The quality of the included studies was evaluated using Law and MacDermid's Appraisal for Clinical Measurement Research Reports, and psychometric properties of play instruments were evaluated using Terwee's checklist while the clinical utility is extracted from each instrument. Initial search identifies 1,098 articles, and only 30 articles were included in the final analysis, extracting 8 play instruments. These instruments were predominantly practiced in the Western culture, which consists of several psychometric evidences. The Revised Knox Preschool Play Scale is considered the most extensive and comprehensive play instrument for extrinsic aspect, whereas the Test of Playfulness + Test of Environmental Supportiveness Unifying Measure is a promising play instrument for intrinsic aspect on play, where both instruments utilize observation. My Child's Play is a potential questionnaire-based play instrument. However, the current development of play instruments in the occupational therapy field is immature and constantly evolving, and occupational therapists should exercise good clinical reasoning when selecting a play instrument to use in practice.

## 1. Introduction

Occupational therapy for children is found as one of the largest practice areas globally [1]. For children, play is the most important occupation that dominates their use of time. Play can be one of the therapeutic goals and can be used as a medium of intervention, which helps to improve an individual's functional performance [1–4]. Play was found to be beneficial for biological, physical, mental, and social development [5]. In general, play is a learning process that equips children with necessary physical, psychological, cognitive, and social skills to facilitate normal development for typical children [6]. Therefore, selecting the right play activities as a means or as an end is important to bring the optimal outcome of children.

Using standardized play assessment can facilitate practitioners in identifying appropriate play activities to be set either as a goal or as a medium of intervention. However, utilization of standardized occupational therapy play instrument even as a research outcome is limited either on occupational therapy intervention [7] or on play-based intervention [2, 3]. An overview of reviews found no study that systematically identifies and investigates standardized occupational therapy instruments on play [8]. Several review studies were found during the literature search but were not in a systematic format. Stagnitti [6] listed three instruments: Knox Preschool Play Scale (and all its variation), Test of Playfulness, and Play History; however, the study was not systematically searched to identify any other play-based instruments. Sturgess [9] suggested several play instruments; however, only Play History and Preschool Play Scale were identified as occupational therapy-based instruments. Two reviews [10, 11] investigated functional assessments for children, and both identified that only the McDonald Play Inventory was used as an instrument tool for play. The limitation of the two reviews was the searching was limited to one journal platform. The absence of comprehensive review study as a guideline will hamper occupational therapy practitioners to efficiently use an appropriate play instrument and to plan an appropriate intervention.

Psychology, speech therapy, physiotherapy, and special education are other disciplines that have interest on play other than occupational therapy. Several instruments were developed by other professions, and several reviews investigated the psychometric properties of these instruments [12–14]. However, each discipline observed each aspect differently. Occupational therapy evaluates play itself, while other professions utilized play activity as a medium to evaluate a particular component [15]. For example, psychologists observe play to specifically evaluate the cognitive function and determine cognitive or social capacity [13, 14], and physiotherapists observe play to evaluate the physical capacity of children [12]. In addition, the only instrument-focused systematic review [12] investigated play-based assessment and not play assessment. Play-based assessment utilizes play activity but evaluates nonplay aspects, such as motor or cognitive functions, whereas play assessment evaluates play for the sake of play.

A study found that occupational therapists used various types of assessments to evaluate play, but some are not purported for play [16]. For example, majority of occupational therapists used Vineland Adaptive Behavior Scale and Battelle Developmental Inventory that evaluate adaptive behavior and general physical, cognitive, and social development in an intention to assess play. This may result in misled judgement on the intervention planning; there is evidence where play is used to elicit improvement in other areas, such as fine motor skills and cognitive function [16, 17]. Therefore, difference on the philosophical foundation of instruments may hinder occupational therapists to efficiently conduct the evaluation and interpret the findings effectively for the purpose of play.

Kuhaneck and colleagues [16] indicated a decreasing trend of using play instrument among occupational therapists. Several reasons were mentioned such as lack of knowledge on available play instruments and lack of continuing education on the existing play instruments. Lynch et al. [18] in their survey found a similar finding where occupational therapists considered play important but indicated lack of education either from research, theory, evaluation, or intervention that contributed to challenges in applying play-centered practice. Meanwhile, Wadley and Stagnitti [19] found that occupational therapists and teachers do appreciate the importance of play for children; however, parents' and family members' understanding on the therapeutic value of play is limited and does not consider play the main goal for the children's functional outcome. Using standardized assessment is part of evidence-based practice [20], enhances the confidence, and strengthens communication and message delivery [21] on the importance of play. Therefore, a systematic review should be conducted to gather play assessments relevant for use in occupational therapy practice to inform the practitioners on the available instruments, enhance evidence-based practice, and select the best instrument for efficient communication medium with clients.

## 2. Materials and Methods

### 2.1. Study Objective

This systematic review was registered on INPLASY (Registration Number: 202040156) and PROSPERO (CRD42020170370). The aim of this review is to identify and gather clinimetric evidence of play instruments developed by occupational therapists. Clinimetric refers to the evidence of psychometric properties (i.e., validity and reliability) and clinical utility of an instrument [22].

### 2.2. Study Identification

A systematic search was conducted on six electronic databases, namely, Academic Search Complete, CINAHL, MEDLINE, Psychology and Behavioral Science Collection, Scopus, and ASEAN Citation Index. Keywords were generated by discussion among authors and reviewing previous literatures. The following keywords were used: (“play” OR “play-based” OR “playthings”) AND (“evaluation” OR “assessment” OR “measurement” OR “battery” OR “test” OR “instrument”) AND (“validity” OR “reliability” OR “sensitivity” OR “precision” OR “specificity” OR “responsiveness” OR “psychometric”) with slight variation. Boolean operators, parenthesis, truncation, and wildcards were used whenever appropriate. For ASEAN Citation Index, only the word “play” was keyed in as the limited function of the search engine that does not allow for search string to be implemented. As the search number was overwhelming, restriction was imposed on keywords existent only in the title for play-related keywords. The search was conducted on 21 January 2020.

Manual search was conducted by screening the reference list of the included study. In addition, the identified instruments were searched for its original article. An innovative method using the “cited by” option in Google Scholar was performed on all original and included articles to allocate more potential articles [23]. Relevant citations were then selected, and the screening process was conducted for eligibility.

### 2.3. Eligibility Criteria

Each retrieved study was evaluated for its eligibility according to the following inclusion and exclusion criteria. The inclusion criteria were (i) study on the instrument for leisure type of play (not competitive play or sports), (ii) instrument generally evaluating play, (iii) study investigating the psychometric property of the instrument, (iv) the instrument used solely on play (not part of a multidimensional instrument), and (v) the instrument relevant for the use of occupational therapy. The last criteria were determined by scrutinizing the instruments found either developed or involved occupational therapist by reviewing the authors of the instrument's original study. Exclusion criteria were (i) not a primary study (i.e., review and editor note), (ii) no full text available, (iii) full text is not available in English, (iv) grey literature (e.g., thesis, book, and conference), and (v) nonpeer review journal article.

### 2.4. Study Selection

Duplicates were initially removed before the screening process. The first author screened the title for eligibility according to the predetermined criteria, followed by independent screening of the abstract and full text by both authors. The preconsensus agreement was calculated by comparing the final accepted articles between the two authors. Any disagreements were resolved through discussion between the two authors until consensus was achieved.

### 2.5. Data Extraction and Analysis

Included articles in the final analysis were narratively analyzed. Each article is extracted for study objective, study design, instrument investigated, number and characteristics of raters, number and characteristics of participants, country of the study, and findings on psychometric property. Extracted play instruments were then identified on its clinical utility focused on the application and administration aspects.

### 2.6. Quality Appraisal of the Study

Two quality assessment tools were used. The quality of each article is assessed using a quality appraisal evaluation form by Law and MacDermid [24]. Terwee's checklist [25] is used to evaluate the pool of psychometric evidence on each instrument found. Although the COnsensus-based Standards for the selection of health status Measurement Instruments (COSMIN; [26]) is considered the gold standard to evaluate the quality of the assessment tool instrument, however, it has several limitations to be used in this systematic review. First, the COSMIN was specifically developed to assess articles demonstrating patient-reported outcomes of health measurement instruments, which might not be suitable for some occupational therapy measurement tools such as play instruments that are complex, varied in terms of administration procedures, involved observation or proxy for rating, and comprised environmental and ecological elements [17, 27, 28]. Second, while the validity of the COSMIN is adequate [29], the reliability of the COSMIN through kappa analysis was poor [30]. Therefore, the use of Law and MacDermid's form and Terwee's checklists is better suited for this study.

Quality Appraisal for Clinical Measurement Research Reports Evaluation Form [24] is a 12-item checklist evaluating the quality of psychometric study on five domains that are research question, design, measurements, analyses, and recommendations. Each item on this form is assigned a score of 0–2 (2, best practice; 1, acceptable but suboptimal practice; and 0, substantially inadequate or inappropriate practice). Only item 6 can be denoted as N/A (not applicable) because it relates to the longitudinal type of study (i.e., test-retest). The total score is calculated by adding all scores from each item and then converted to a percentage. Higher score indicates better quality. The form was developed by rehabilitation experts from occupational therapy and physiotherapy backgrounds and has excellent interrater reliability [31–37] that has been used in environment-based instruments [28]. Quality assessments were administered by both authors and verified through discussion.

The Terwee checklist is an assessment tool to determine the quality of psychometric properties of the instrument [25]. For that purpose, studies were grouped based on the instrument described, and a summary of psychometric properties of each instrument was then prepared according to eight categories, namely, (i) content validity, (ii) internal consistency, (iii) criterion validity, (iv) construct validity, (v) reproducibility (agreement and reliability), (vi) responsiveness, (vii) floor or ceiling effect, and (viii) interpretability. Each instrument was then assessed against the quality criteria and rated according to four categories: positive (i.e., +), which means having a desired outcome with robust methodology; intermediate (i.e., ?), which means having a desired outcome with less robust methodology; poor (i.e., -), means having an undesired outcome or having poor methodology; and no information available (i.e., N/A). When two or more studies investigated the same property, the highest quality score for that item was recorded.

## 3. Results

**Figure 1:**
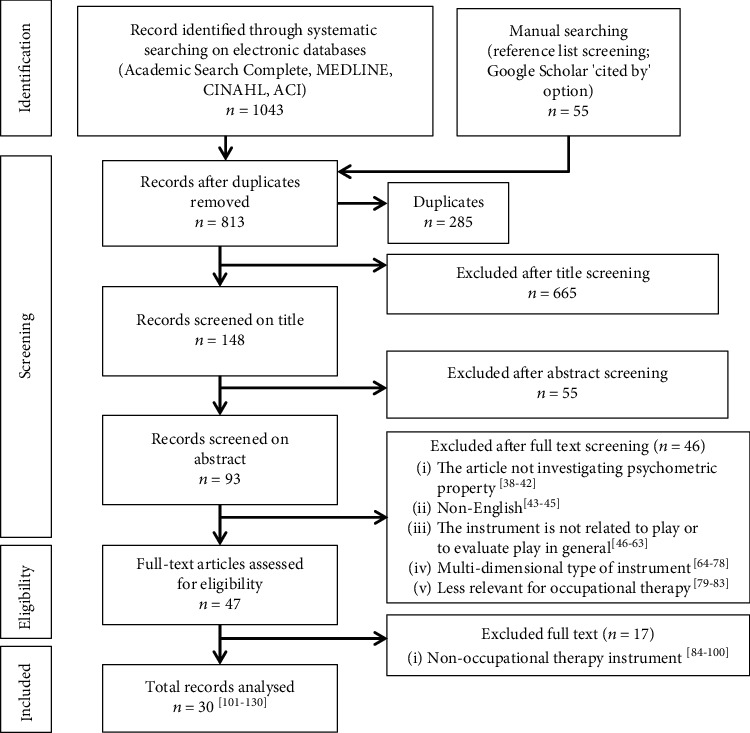
Screening process.

A total of 1,098 articles were retrieved; 1,043 were obtained from the electronic database search, and another 55 were later identified from the reference list of the included studies and list of relevant literature found using Google Scholar's “cited by” option. Ultimately, as shown in Figure 1, 63 [38–100] articles were excluded during the full-text screening and 30 [101–130] individual studies were selected after the screening process by the two authors (preconsensus agreement on accepted full text: 79.4%). The description of each included individual study and its psychometric report is presented in Table 1.

Quality of individual studies was measured using Law and MacDermid's Quality Appraisal Tool, and the result is presented in Table 2. Overall, studies have the median score quality of 65.5% (range, 45–86%).

Eight original occupational therapy play instruments were extracted from the 30 included articles. The included instruments are (i) Child-Initiated Pretend Play Assessment (ChIPPA; including Indigenous Play Partner Scale), (ii) Revised Knox Preschool Play Scale (Knox PPS), (iii) McDonald's Play Inventory (MDPI), (iv) My Child's Play (MCP), (v) Play Assessment for Group Setting (PAGS), (vi) Playform, and (vii) Play History Interview (PHI) and Test of Playfulness (ToP, including Test of Environmental Supportiveness (TOES) and ToP-TOES Unifying Measure (T-TUM)). One occupational therapy instrument—Play Skills Inventory [38]—was found but was not included as there is no published journal article that investigated its psychometric property. Some of the instruments were published only once (i.e., McDonald's Play Inventory, My Child's Play, Playform, Play History Interview, and Play Assessment for Group Setting), whereas some were reported in several articles in different occasions (i.e., Knox PPS, ToP+TOES, and I-ChIPPA). Further analysis on the excluded full text articles was also conducted to identify available play instruments and listed in Box 1. However, those instruments are presented for information purpose and not to be included for analysis as they are nonoccupational therapy play instruments.

Instruments found usually investigated for concurrent and construct validity and interrater and test-retest reliability. Some instruments such as Knox PPS have been investigated on the same psychometric properties (e.g., interrater reliability and concurrent validity) over time. Homogeneity on the study location was identified where majority of the instruments have been investigated at the origin country. Most of the origin countries are Caucasian-dominant countries that are heavily influenced by the Western culture. The summary on psychometric evidences of each instrument extracted from individual studies is presented in Table 3.

Several instruments are observation-based (i.e., ChIPPA, ToP, Knox's PPS, and Play Assessment for Group Settings) and evaluated by observing the children in play activities either in real situations or recorded videos, while some are perception-based by rating a questionnaire (i.e., McDonald's Play Inventory, My Child's Play, and Playform), and another is subjective-based instrument that retrieves information from a qualitative interview (i.e., Play History Interview). Most instruments focused on extrinsic elements, such as developmental, behavior and attitude, and skills and performance, except for ToP that views the intrinsic factor (e.g., motivation) of play.

In terms of availability, majority of the instruments are not commercially available. Only the ChIPPA, Knox PPS, and ToP are made commercial. However, ChIPPA is the costliest, whereas the other two are at an affordable range. For the other instruments, contacting the author to obtain the original instrument may be required. The utility description of each instrument is presented in Table 4.

## 4. Discussion

This review found various play instruments where only a small number were developed by an occupational therapist. Some of the instruments were also mentioned and described in the previous reviews [6, 9–11], and some of them are newly identified. Clemson and colleagues [131] suggested that an instrument should have at least evidence on content validity and interrater reliability. Conversely, Prinsen et al. [132] specified that an instrument should at least establish a psychometric evidence on content validity, followed by the internal structure of the instrument (i.e., structural validity, internal consistency, and cross-cultural validity). The instruments found in this review had at least basic psychometric property. However, when compared to other function-based instruments for children [8], the play instruments found have limited number of psychometric properties investigated. The psychometric investigation of the instruments was mostly around interrater reliability and content validity. Therefore, investigation on the other psychometric properties is warranted. In addition, the methodological quality of the studies is moderate. Aspects such as sample characteristics of the population type, number, size of the client participants and assessor participants, and generalizability can further be improved.

Several occupational therapy play instruments are recommended based on occasion. The Revised Knox Preschool Play Scale is considered the gold standard for occupational therapy play assessment and suitable to be used to evaluate extrinsic aspect of play. The Revised Knox Preschool Play Scale is an all-rounder that covers an extensive number of domains. Moreover, it is the most common play instrument tool used by occupational therapists and considered easy to administer [16]. In addition, the instrument is accepted across discipline such as psychologist and speech therapist, thereby becoming a good communication tool between disciplines. The Test of Playfulness and its extension, the Test of Environmental Supportiveness, are a unique instrument that can be used across the widest age range and evaluate the internal element of play, such as motivation; however, the latest innovation of instruments, known as ToP-TOES Unifying Measure (T-TUM), is a promising play instrument. The Revised Knox Preschool Play Scale and Test of Playfulness utilized observation. Observation provides qualitative findings that are useful and valuable for practitioners to complement their evaluation finding on the quantitative outcome [17]. However, using observational instruments may be less favorable for busy practitioners and on setting with various constrains [6]. Therefore, a questionnaire-based instrument is sought, and the My Child's Play instrument can be potentially used for this purpose. The selection of those instruments over the others considers the balance on the clinimetric properties. Psychometric evidence only does not guarantee an instrument application in practice; clinical utility of the instruments also plays a crucial role [28, 133]. Nevertheless, play instruments in occupational therapy remain immature and evolving; therefore, several potentials and opportunities are available to explore a new instrument development or improve the currently available instruments.

Play is an activity that may be influenced by geosociocultural environment surrounding a person [6, 17]. Cultural value may impose a meaning on an activity, including play. For example, a study by Dender and Stagnitti [107] found that indigenous children appreciate animal toys that resemble their culture compared to the common commercialized farm animal toys. Moreover, children struggle to perform pretend play using the given “scrap” materials because the material is foreign to their culture. In addition, the indigenous children also have difficulty to play alone as mostly the play activity happen in pair or group in the indigenous culture. Most instruments were developed in a developed and Western-influenced country, such as Australia and the United States. Thus, using an instrument developed in one culture to another distinct cultural group may unfairly disadvantage the latter one [134]. The accuracy of an instrument may be reduced; however, improper remedial of the instrument to suit another cultural need may affect the validity of the instrument where it cannot inform any group evaluated. The cross-cultural investigation on functional instrument tools for children is emphasized and warranted [135]. Limited investigation on cross-cultural validity has restricted the widespread applicability of play instruments internationally. Therefore, the usability of play instruments can widely be investigated among cross-countries.

Authorship bias may exist from included studies, and this may compromise the report quality of the article. Involvement of the developer or creator of the instrument in the included studies may have contributed toward bias on the discussion of findings such as emphasizing on positive arguments and suppressing negative outcomes [136]. Only the Knox PPS was found to minimize the impact of the authorship bias; all included studies on Knox PPS have little to no involvement of the original developer of the instrument. Involvement of the original developer has its benefits such as encouraging the promotion and research on the particular instrument but may be associated with challenges such as the aforementioned bias. Hence, any conflict of interest and funding disclosure should be properly addressed [137]. Readers should cautiously assess the information to ensure reaching a neutral decision.

The clinical utility is another aspect that should be considered besides the psychometric property of an instrument. Although this review did not extensively search for clinical utility, majority of instruments embedded a report on the clinical utility of instruments such as the ChIPPA (see Pfeifer et al. [120]). Some instruments such as Test of Playfulness reported the clinical utility in a separate publication [138]. Clinical utility aspects that warranted attention from researchers are on appropriateness (e.g., importance of clinical decision-making and impact on the existing treatment process), accessibility (e.g., cost-effectiveness, availability, and support by peer-professionals and organizations), practicability (e.g., suitability across settings and professional and training requirement), and acceptability (e.g., ethical, social, or psychological concern) [139]. Most of the publications reported the duration of administration and training requirement. However, explicit clinical utility should be reported together with the psychometric property publication of the instruments. This will increase the relevancy of instruments to be used by practitioners.

Majority of play instruments focused on preschool and school-aged children; limited for newborns, infants, and toddlers; and negligible for adolescents. While play is known as the dominant activity for children, its essence is available across lifespan [6, 140, 141]. The neglected populations are somewhat denied on their right to play. Other disciplines such as psychology have carefully considered this approach. For example, the Fair Play Questionnaire is a generic instrument that evaluates the social and ethical opportunity of adolescents in play participation especially in structured play [86]. Other studies investigated instruments to evaluate the playfulness among older people [83, 99]. As the developmental stages become more mature such as adolescents and adults, play concept usually inhibited and replaced with leisure [140], and this is where play evaluation is not a priority. For example, Henry [60] and Trottier et al. [63] examined the instrument on leisure aspect of adolescents as this concept becomes the main focus compared to play during this stage of the lifespan. However, play element should continue to be investigated across the lifespan.

### 4.1. Implication to Practice

Play has been argued as a complex construct and influenced by relative multidimensionality. Only a study by Rigby and Gaik [121] investigated the stability of measuring play in several settings (i.e., home, community, and school) which found that it may influence the playful experience but not exclusive to the specific type of setting. For example, one child may experience the highest level of playfulness at home and lowest at school, whereas another child may experience otherwise. Another study by Kielhofner et al. [41] showed that environmental setting and involved personnel significantly contribute toward the play quality. This warranted an attention to consider the environment as a mediating factor. Hence, among the play instruments found in this review, T-TUM has successfully addressed the issue of environmental effects but may require further investigation. On the other hand, a study by Hyndman et al. [100] indicate that play perception varies between days in a week and varies on happiness level perception before and after the play. This aspect was not extensively investigated in any occupational therapy play instrument. This information should be crucially considered when conducting play assessment to ensure consistent outcome and interpretation.

In practice, practitioners require an instrument that can provide information on extensive number of aspects, requires minimal training and low administrative burden, and is easy to interpret [21, 142]. However, occupational therapy practitioners should consider both characteristics (i.e., skills) and quality traits (i.e., enjoyment) on play either during the evaluation or intervention. Planning a play activity as intervention may support or inhibit the progression of clients depending on the appropriateness of planning. Using an appropriate standardized assessment is one of the ways to facilitate proper and evidence-based planning [21]. Having a good standardized assessment may provide confidence to practitioners in rationalizing the service [19, 20]. However, play is associated with various ambiguities, and the current development of existing instruments on play is limited to one small part of play as mentioned by Bundy [17]—“reducing play to skills” (p. 99)—that is unable to provide a holistic picture on the client's play condition. To address the current limitations, practitioners should exercise good clinical reasoning skills. Synthesizing the objective outcome (i.e., standardized assessment result) with clinical reasoning (i.e., values and belief) will strengthen the planning that benefits the client [6, 17, 143]. Therefore, practitioners should combine findings from the instrument with clinical reasoning for a better service.

### 4.2. Limitation and Recommendation

This systematic review has several limitations to be noted. First, articles included were only obtained from journal publications, and therefore, evidence on psychometric properties of the instrument may not be comprehensive. Several psychometric evidences such as content validity may be available in the manual instrument book such as ChIPPA [144]. Several instruments are only available in grey literature format that is not captured during the review search. For example, the Kid and Preteen Play Profile can be found in a book [145]. Second, the review only included publication in English language. Several articles found in this review were in foreign languages but excluded due to the limited ability to understand the articles. This is associated with disadvantages involving instruments that provide more psychometric evidences, especially on cross-cultural applicability. Third, some psychometric properties of the instrument are briefly reported as a small part of the original study (see, for example, Okimoto et al. [130]), which compromise reporting on the quality and inability to provide a detailed description on the psychometric evidence. Fourth, the use of Terwee's checklist is still not comprehensive enough to illustrate the available type of psychometric properties. Even the COSMIN taxonomy [132] does not provide the available extensive type of validity and reliability. According to Law and MacDermid [24], more than 25 types of validities and reliabilities were found. Therefore, future research may try to investigate other types of validity and reliability that can be added on the number of psychometric evidence of the instruments besides the existing ones. Nevertheless, this review can provide a comprehensive guideline for practitioners to select an appropriate play instrument in practice.

## 5. Conclusions

Several play assessments are available for occupational therapists used in practice. Outcome from standardized play instrument may convince stakeholders and clients to change their perception on play as a main goal for children rehabilitation. However, the current development of play instruments is immature and constantly evolving. Available instruments are constantly developed and continue to be improved. Nevertheless, several instruments such as the Revised Knox Preschool Play Scale are suitably used as a comprehensive play evaluation for extrinsic perspective of play. The Test of Playfulness + Test of Environmental Supportiveness Unifying Measure is promising in evaluating intrinsic perspectives of play. As both instruments utilized an observation approach, My Child's Play is a potential instrument for a questionnaire-based reported outcome. However, practitioners need to consider several aspects such as client's needs, support, and facility condition and exercise good clinical reasoning when selecting an instrument for use.

## Figures and Tables

**Box 1:**
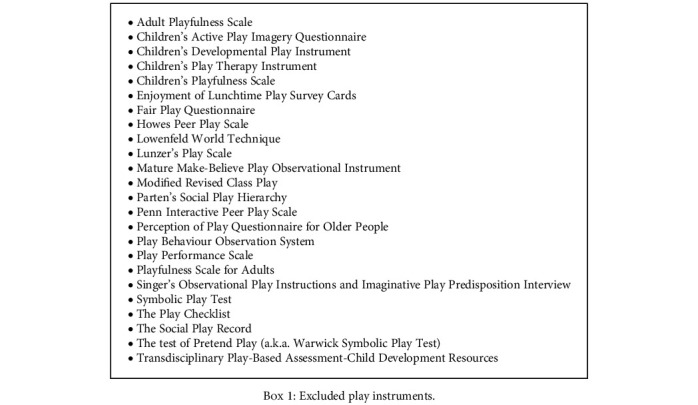
Excluded play instruments.

**Table 1:** Characteristic and psychometric reporting of individual studies.

Author	Year	Instrument	Objective	Study design	Country	Participant	Rater	Finding
Dender & Stagnitti [108]	2017	IPPS	To explore the content and cultural validity for social aspect of the instrument	Qualitative	Australia	6 pairs of indigenous children (i.e., 12 children) 14 community elders and mothers	—	The extension instrument is culturally accepted and nonjudgmental.
Golchin et al. [109]	2017	ChIPPA	To establish the reliabilities, content, and cross-cultural validity of the translated Persian version of the instrument	Cross-sectional (validity) Cohort (reliabilities)	Iran	5 occupational therapists 31 typical children	2 researchers	Internal consistency is *α* = 0.752. Reliability is excellent for intrarater (ICC = 0.99), interrater (ICC = 0.98) and moderate to strong for test-retest (ICC = 0.69–0.99). Content validity is strong (CVR = 0.81–1.00).
Stagnitti & Lewis [129]	2015	ChIPPA	To investigate the predictive validity of the instrument on semantic organization and narrative retelling skills using SAOLA	Cross-sectional	Australia	48 typical and at risk of learning difficulty children	3 examiners	The instruments predicted 23.8% of semantic organization and 18.2% of narrative retelling skills.
Dender & Stagnitti [107]	2011	I-ChIPPA	To investigate the cultural appropriateness of the adapted instrument and its reliability	Qualitative Cross-sectional	Australia	23 indigenous Australian children (i.e., 12 pairs)	4 indigenous children	Cultural adaptation is satisfactory. The toys were found to be gender-neutral (*p* > 0.05). Overall, interrater reliability on toy use is moderate (ICC = −0.33–1.00).
Pfeifer et al. [120]	2011	ChIPPA	To establish the cross-cultural validity and reliability of the translated Portuguese version of the instrument	Cross-sectional	Brazil	14 typical children	1 occupational therapy student and 1 supervisor	Validity is established where the play material and duration are appropriate with the Brazilian context. Intrarater reliability is good (*r* = 0.90–0.97). Interrater reliability is moderate (*r* = 0.13–0.76).
McAloney & Stagnitti [117]	2009	ChIPPA	To investigate the concurrent validity of the instrument	Cross-sectional	Australia	53 typical children	1 researcher	Significant negative correlation was found between play and social.
Uren & Stagnitti [128]	2009	ChIPPA	To investigate the construct validity of the instrument	Cross-sectional	Australia	41 children of typical or minor disabilities	5 teachers	There is probable evidence on construct validity of the instrument Penn Interactive Peer Play Scale (PIPPS) and Leuven Involvement Scale for Young Children (LIS-YC) where several components were significantly moderately correlated.
Swindells & Stagnitti [127]	2006	ChIPPA	To investigate the construct validity of the instrument	Cross-sectional	Australia	35 typical children	2 researchers	Interrater reliability is strong (*k* = 0.7). There is probable evidence on construct validity of the instrument with Vineland Social-Emotional Early Childhood Scales; overall not significant but certain aspects were found significantly correlated.
Stagnitti & Unsworth [124]	2004	ChIPPA	To establish test-retest reliability of the instrument	Longitudinal	Australia	38 typical and developmental delay children	1 researcher	Test-retest reliability is moderate to strong (ICC = 0.57–0.85).
Stagnitti et al. [125]	2000	ChIPPA	To ascertain the discriminant validity and interrater reliability of the instrument	Cross-sectional	Australia	82 typical and preacademic problem children	3 occupational therapists	Interrater reliability is excellent (*k* = 0.96–1.00). Discriminant validity is established (*p* < 0.001)
Sposito et al. [123]	2019	Knox PPS	To verify the reliabilities of the Brazilian version of the instrument	Cross-sectional	Brazil	135 typical children	2 undergraduate occupational therapy students	Overall, the internal consistency is good (*α* = 0.48–0.95). Overall intrarater reliability (*k* = 0.18–0.99) is reported to be moderate to excellent and interrater reliability (*k* = −0.03–0.71) is moderate.
Pacciulio et al. [119]	2010	Knox PPS	To investigate the reliability and repeatability of the Brazilian version	Cohort	Brazil	18 typical children	2 examiners (one is the researcher; no further detail)	Strong intrarater correlation between the two occasions (*r* = 0.87–1.00). Strong interrater correlation between the two examiners (*r* = 0.78–0.99).
Lee & Hinojosa [116]	2010	Knox PPS	To establish the interrater and concurrent validity of the revised version of the instrument	Cross-sectional	United States of America	61 children with autism	2 researchers	Interrater reliability is excellent (ICC = 0.94) and construct validity with VABS is moderate (*r* = 0.52, *p* < 0.01).
Jankovich et al. [112]	2008	Knox PPS	To establish the interrater and construct validity of the revised version of the instrument	Cross-sectional	United States of America	38 typically developing children	2 occupational therapy students	Interrater agreement is high (81.8%–100%). Higher agreement was achieved on observation of older than younger children. Construct validity showed higher agreement between chronological and average play age for older than younger children.
Harrison & Keilhofner [111]	1986	Knox PPS	To determine the interrater and test-retest reliability and validity of the original instrument	Cross-sectional (interrater; concurrent validity) Longitudinal (test-retest)	United States of America	60 disabled preschool children	3 observers (detail not mentioned)	Overall interrater reliability is substantial (ICC ≈ 0.67). Overall test-retest correlation is strong (*r* = 0.55–0.97). Concurrent validity indicates that the instrument correlates moderately with Parten's Social Play Hierarchy (*k*_Tau_ = 0.60–0.64) and Lunzer's Scale on Organization of Play Behavior (*k*_Tau_ = 0.50–0.89). The instrument correlated moderately with age (*r* = 0.01–0.91) for disabled children but strongly with typical children.
Bledsoe & Sheperd [102]	1982	Knox PPS	To determine the inter-rater, test-retest reliability and validity of the revised instrument	Cross-sectional (inter-rater; concurrent validity) Longitudinal (test-retest)	United States of America	90 typical children	2 researchers cum observers	Overall, the inter-rater and test-retest yielded satisfactory correlation. Concurrent validity indicates that the instrument correlates moderately with Parten's Social Play Hierarchy and Lunzer's Scale on Organization of Play Behavior. The construct validity indicates that the instrument is correlated strongly with age.
McDonald & Vigen [118]	2012	McDonald Play Inventory	To examine the content, construct and discriminative, validity, internal consistency, and test-retest reliability of the instrument	Cross-sectional (validities, internal consistency) Longitudinal (test-retest)	United States of America	124 children 17 parents	Self/proxy-rating 7 children (test-retest)	Content validity is overall moderately correlated between items. Construct validity found that the instrument can discriminate between typical and disabled children. Concurrent validity between parent-child rating has low to moderate correlation (*r* = 0.04–0.49). Test-retest was strongly correlated (*r* = 0.69–0.82) between two time points. Internal consistency: *α* = 0.79–0.84.
Schneider & Rosenblum [122]	2014	My Child's Play	To describes the development, reliability, and validity of the instrument	Cross-sectional	Israel	334 mothers	—	Concurrent validity with Parent as a Teacher Inventory is fair (*r* = 0.33; *p* < 0.001). Factor analysis established construct validity (*α*s = 0.63–0.81). Gender (girls>boys) and age were significantly different in score. Internal consistency: *α* = 0.86.
Lautamo & Heikkilä [113]	2011	PAGS	To investigate the interrater reliability of the instrument	Cross-sectional	Finland	78 typical and atypical children	12 professionals (teachers, occupational therapist, physiotherapist)	MFR on expected agreement (44.1%) and the observed agreement (50.8%) with Rasch kappa of 0.12.
Lautamo et al. [115]	2011	PAGS	To evaluate the validity of the instrument for use with children with language impairment over typical children	Cross-sectional	Finland	156 typical and language impairment children	Proxy-rating (teachers, special education teachers, nurses, physiotherapist, occupational therapist)	The analysis found significant difference between the two groups, but 80% of the items are considered stable.
Lautamo et al. [114]	2005	PAGS	To determine the construct validity of the instrument	Cross-sectional	Finland	93 typical and atypical children	Proxy-rating (teachers, special education teachers, nurses, occupational therapist)	The construct validity of the instrument is established by internal scale validity, and person response validity achieved strong goodness of fit value.
Behnke & Fetkovich [101]	1984	Play History Interview	To determine reliability in terms of interrater and test-retest and validity of the Play History Interview	Cross-sectional (interrater; concurrent validity) Longitudinal (test-retest)	United States of America	30 parents with nondisabled or disabled children	2 researchers cum raters	Concurrent validity with Minnesota Child Development Inventory is overall moderate to strong. Known-group validity is able to discriminate between disabled and nondisabled children (*p* < 0.01). Interrater reliability is moderate to strong while test-retest has fair to strong correlation.
Sturgess & Ziviani [126]	1995	Playform	To explore the consistency on rating the instrument between three groups of rater	Cross-sectional	Australia	13 children 13 parents 1 teacher	—	Qualitatively, the rating between the three groups is relatively similar; parents scored slightly more positive than the children, but teachers are the most positive.
Bundy et al. [106]	2009	T-TUM	To investigate the translatability of the instrument to practice known as T-TUM (ToP+TOES Unifying Measure)	Cross-sectional	United States of America	265 atypical children	—	At least 92% of the outcomes were within the limit for goodness of fit. The reliability enhanced to *α* = 0.96 for T-TUM.
Brentnall et al. [103]	2008	ToP	To evaluate the validity of instrument rating over different lengths and point of time	Cross-sectional	United States of America	20 typical children	3 researchers cum raters	Different time points have no significantly different observation outcome (*p* = 0.204) but significantly different than longer observation time (*p* < 0.001) but provide no added information. Longer observation time has poorer test-retest value (ICC = 0.033) compared to shorter time (ICC = 0.408–0.668).
Rigby & Gaik [121]	2007	ToP	To investigate the stability of the instruments over three different settings	Cohort	United States of America	16 children with cerebral palsy	1 researcher	The score showed significant difference across the three settings (i.e., home, community, and school) (*p* < 0.05). The children are most playful at home and least playful at school.
Hamm [110]	2006	ToP + TOES	To examine the validity and reliability of the instruments with children with and without disabilities	Cross-sectional	United States of America	40 children with and without disabilities	2 trained raters	Interrater agreement is 100%. Item response validity is 100%, and internal scale validity is 100%. There is less playfulness but higher correlation of the instrument with children with disabilities than without disabilities.
Bronson & Bundy [104]	2001	ToP + TOES	To evaluate the validity of the two instruments	Cross-sectional	United States of America	160 children with and without disabilities	10 raters (not specified)	The reliability is acceptable: *α* = 0.77. TOES construct validity is acceptable (94% fit). The environment (i.e., TOES) is correlated significantly with playfulness (i.e., ToP) (*r* = 0.401; *p* = 0.01). The TOES has significant difference between typical and disabled children (*z* = 2.96; *p* = 0.05).
Bundy et al. [105]	2001	ToP	To investigate the construct and concurrent validity and interrater reliability of the instrument	Cross-sectional	United States of America	124 children (typical and special education) in total	26 occupational therapists	Construct validity explained 93% of the items unidimensional construct on playfulness. Concurrent validity with Children's Playfulness Scale was found to be moderate (*r* = 0.46; *p* < 0.001). Interrater reliability achieved 96% consensus.
Okimoto et al. [130]	1999	ToP	To investigate the reliability and validity of the instrument	Cross-sectional	United States of America	54 videotaped mother-CP-child dyad	3 occupational therapists	The reliability is 97.5% fit within the acceptable range. The instrument was found to be sensitive to change.

ChIPPA: Child-Initiated Pretend Play Assessment; I-ChIPPA: Indigenous ChIPPA; IPPS: Indigenous Play Partner Scale; Knox PPS: Revised Knox Preschool Play Scale; PAGS: Play Assessment for Group Setting; ToP: Test of Playfulness; TOES: Test of Environmental Supportiveness; T-TUM: ToP-TOES Unifying Measure.

**Table 2:** Quality assessment on each included study using Law and MacDermid [24] tool.

Studies	Instrument^◊^	Evaluation criteria^†^ (score: 2 = good, 1 = moderate, 0 = poor, N/A = not applicable)												Total score (%)
		Item 1	Item 2	Item 3	Item 4	Item 5	Item 6	Item 7	Item 8	Item 9	Item 10	Item 11	Item 12	
Dender & Stagnitti [108]	IPPS	2	2	2	1	1	N/A	2	2	2	2	0	2	82
Golchin et al. [109]	ChIPPA	2	2	1	2	2	1	2	1	1	2	2	1	79
Stagnitti et al. [125]	ChIPPA	2	2	2	1	0	N/A	2	2	2	2	0	2	77
Stagnitti & Lewis [129]	ChIPPA	2	2	2	0	0	N/A	2	2	2	2	0	1	68
Uren & Stagnitti [128]	ChIPPA	2	1	2	0	0	N/A	2	2	2	2	0	1	64
Swindells & Stagnitti [127]	ChIPPA	2	0	2	0	0	N/A	2	2	1	2	2	1	64
Stagnitti & Unsworth [124]	ChIPPA	1	1	1	0	0	2	2	2	2	2	1	1	63
Dender & Stagnitti [107]	I-ChIPPA	1	2	2	1	0	N/A	1	1	1	2	0	2	59
Pfeifer et al. [120]	ChIPPA	2	1	1	1	0	N/A	2	2	1	1	0	2	59
McAloney & Stagnitti [117]	ChIPPA	1	1	2	0	0	N/A	2	2	2	1	1	1	59
Sposito et al. [123]	Knox PPS	2	2	1	1	2	N/A	2	1	1	2	0	2	73
Jankovich et al. [112]	Knox PPS	2	2	2	1	0	N/A	2	2	2	1	0	2	73
Lee & Hinojosa [116]	Knox PPS	2	2	2	1	0	N/A	1	1	1	2	0	2	64
Bledsoe & Sheperd [102]	Knox PPS	2	2	2	2	1	0	1	1	2	1	0	1	63
Harrison & Keilhofner [111]	Knox PPS	1	2	1	2	0	1	1	1	2	1	0	1	54
Pacciulio et al. [119]	Knox PPS	2	1	1	1	0	N/A	1	1	1	1	0	1	45
McDonald & Vigen [118]	McDonald Play Inventory	2	2	1	2	1	0	2	1	2	1	0	1	63
Schneider & Rosenblum [120]	My Child's play	1	1	1	2	1	N/A	2	0	1	2	1	2	64
Lautamo et al. [115]	PAGS	2	2	2	0	1	N/A	2	2	2	2	2	2	86
Lautamo & Heikkilä [113]	PAGS	2	1	2	0	1	N/A	2	2	2	2	1	2	77
Lautamo et al. [114]	PAGS	2	1	2	0	1	N/A	2	1	2	2	2	2	77
Behnke & Fetkovich [101]	Play history interview	2	2	2	2	0	0	2	1	2	1	0	2	67
Sturgess & Ziviani [126]	Playform	2	2	1	0	0	N/A	1	2	1	0	0	1	45
Bundy et al. [106]	T-TUM	1	2	2	1	1	N/A	2	2	2	2	1	2	82
Bronson & Bundy [104]	ToP + TOES	2	2	2	2	1	N/A	2	2	1	1	1	2	82
Hamm [110]	ToP + TOES	2	2	2	2	0	N/A	2	2	1	1	1	2	77
Bundy et al. [105]	ToP	2	2	2	2	1	N/A	2	2	1	1	0	2	77
Brentnall et al. [103]	ToP	2	1	2	1	0	2	1	2	2	2	1	2	75
Rigby & Gaik [121]	ToP	2	2	2	0	0	N/A	2	2	1	1	0	1	59
Okimoto et al. [130]	ToP	2	2	1	0	0	N/A	1	1	2	1	0	1	50

^†^Item 1: relevant background on psychometric properties and research question; item 2: inclusion/exclusion criteria; item 3: specific psychometric hypothesis; item 4: appropriate scope of psychometric properties; item 5: appropriate sample size; item 6: appropriate retention/follow-up; item 7: specific descriptions of the measures (administration, scoring, interpretation procedures); item 8: standardization of methods; item 9: data presented for each hypothesis or purpose; item 10: appropriate statistical tests; item 11: appropriate secondary analyses; and item 12: conclusions/clinical recommendations supported by analyses and results. ^◊^ChIPPA: Child-Initiated Pretend Play Assessment; I-ChIPPA: Indigenous ChIPPA; IPPS: Indigenous Play Partner Scale; Knox PPS: Revised Knox Preschool Play Scale; PAGS: Play Assessment for Group Setting; ToP: Test of Playfulness; TOES: Test of Environmental Supportiveness; T-TUM: ToP-TOES Unifying Measure.

**Table 3:** Summary of the quality of psychometric properties of the instruments.

Instrument tool	Terwee checklist [25] (score: + = positive; ? = intermediate; – = poor; 0 = no information available)								
	Content validity	Internal consistency	Criterion validity	Construct validity	Reproducibility		Responsiveness	Floor or ceiling effect	Interpretability
					Agreement	Reliability			
ChIPPA	+^a^, +^b^, +^c^	?^b^	0	?	?, ?^b^	?, ?^a^_,_ ?^b^	0	0	0
Knox's PPS	?	?^d^	0	?	?	+, ?^d^	0	0	0
McDonald Play Inventory	0	?	0	—	0	?	0	0	0
My Child's Play	+	+	0	+	0	0	0	0	0
PAGS	+	0	0	+	0	+	0	0	0
Play History Interview	?	0	0	?	?	?	0	0	0
Playform	+	0	0	0	0	0	0	0	0
ToP + TOES, T-TUM	+^e^, +^f^, +^g^	+^f^, +^g^	0	+^e^, +^f^, +^g^	?^e^, ?^f^	?^e^, ?^f^	0	0	?^f^, +^g^

^a^Brazilian-Portuguese ChIPPA; ^b^Iranian ChIPPA; ^c^Indigenous Play Partner Scale (I-PPS); ^d^Knox's Play Scale; ^e^Test of Playfulness (ToP); ^f^Test of Environmental Supportiveness (TOES); ^g^ToP-TOES Unifying Measure (T-TUM).

**Table 4:** Usability of the instruments.

Instrument	Description	Procedure	Population	Administration	Duration	Scoring	Training requirement	Accessibility
Child-Initiated Pretend Play Assessment (ChIPPA) (i) I-ChIPPA	Assessment of the quality of a child's ability to self-initiate pretend play.	Observation on two play scenarios [15 minutes each, (i) conventional imaginative play using toys, (ii) symbolic play using “junk” materials].	Children age 3–7 years old	Therapist observation	30 minutes	Actions observed were coded and then counted to be translated into raw score. The raw score is then calculated in and transformed into percentage according to norm reference.	Self-learning through manual (75-minute video)	Require purchase
Extension of I-ChIPPA: Indigenous Play Partner Scale (IPPS)	The extension (i.e., IPPS) provides an added evaluation on social aspect in play.	The IPPS added the observation on playing in pair.	For indigenous Australian	Similar to original instrument	Simultaneously with the original evaluation duration	Additional scoring on initiative playing in pair for social context.	Additional reading on the journal article	Part of the original purchase
Knox's Preschool Play Scale (i) Knox Play Scale (ii) Preschool Play Scale (iii) Revised Knox's Preschool Play Scale	The instrument has been evolved over time. The instrument is to evaluate children's developmental play ages.	Consist of 4 dimensions (space management, material management, pretense/symbolic, participation) and 12 categories of play behaviors. 30-minute observation each for inside and outside play.	Children age 0 – 6 years old.	Observation	1 hour	Each category (a.k.a. factor) is scored with either a+ when the behavior was present, a− when the behavior was absent, or NA when no opportunity to observe. The scoring is according to age, and play development is scored by transforming into mean score on factor/dimension.	Not required. Self-training by reading the manual	Require purchase
McDonald Play Inventory	The instrument is to measure play frequency and play style.	The instrument consists of two parts. Part one (i.e., MPAI) has four categories, and part two (i.e., MPSI) has six domains with a total of 80 items.	Children age 7–11 years old	Self-reported (children) Proxy-reported (parents)	15 minutes (without assistance) 20–30 minutes (with assistance)	Each item was scored on a five-point Likert scale. Total score is calculated by summing-up the individual scores for each part.	No training required	May need to contact the author
My Child's Play	The instrument is to measure parent's perception on child' play performance.	The instrument has four categories with a total of 45 items.	Children age 3–9 years old	Proxy-administered (parents)	Not mentioned	Each item was scored on a five-point Likert scale. Total score is calculated by summing-up the individual scores. Higher score indicates better outcome.	No training required	May need to contact the author. Possible to replicate from the article
Play Assessment for Group Settings (PAGS)	The instrument is to evaluate attitude on organized and imaginative play.	The instrument has 38 items and evaluated by observation during play activities in several occasions.	Children age 2–8 years old	Professional rater	No specific duration	Each item was scored on four-point Likert scale. The raw score is totaled from the individual items and then computed and transformed to logits.	No to minimal training (or self-training)	May need to contact the author. Possible to replicate from the article
Play History Interview	A semistructured qualitative questionnaire to identify play experiences, interactions, environments, and opportunities.	Five epochs: sensorimotor, symbolic and simple constructive, dramatic and complex constructive and pregame, games, recreational.	Children age 0–16 years old	Therapist interviewing the caregivers	Not specified	Qualitative response on each epoch—materials (what), action (how), people (with whom), setting (where).	Encourage to be trained	May need to contact the author. Difficult to reproduce from the journal article
Playform (i) Child form (ii) Parent form (iii) Teacher form	The instrument is to evaluate child's play competency.	The instrument has 20 items.	Children age 5–7 years old	Self-administered (children) Proxy-administered	Average 15 minutes (range 10–25 minutes)	Scored each item on three-point Likert scale (“not very well,” “quite well,” “very well”). Total score is by counting the “very well” response.	No training required	May need to contact the author. Unable to reproduce from the journal article
Test of Playfulness (ToP)	The instrument is to evaluate intrinsic and internal component that reflects a child's transaction in a play context.	The instrument has four elements with a total of 29 items.	6 months–18 years old	Therapist observation	15 minutes	Each item was scored on four-point Likert scale. The score is then totaled overall from raw score and converted into a measure score.	Self-training by reading the manual	Require purchase
Extension of ToP: Test of Environmental Supportiveness (TOES)	The instrument is to evaluate the element of environment that influences play.	The instrument has 17 items and focuses on five elements.	15 months–12 years old	Similar to original instrument	Simultaneously with the original evaluation duration	Each item was scored on four-point Likert scale. The score is then totaled overall from raw score and converted into a measure score.	Self-training by reading the manual	Part of the original purchase
