# Corrigendum to “A Systematic Review on Clinimetric Properties of Play Instruments for Occupational Therapy Practice”

**DOI:** 10.1155/2020/9475025

**Published:** 2020-09-05

**Authors:** Muhammad Hibatullah Romli, Farahiyah Wan Yunus

**Affiliations:** ^1^Department of Nursing and Rehabilitation, Faculty of Medicine and Health Sciences, Universiti Putra Malaysia, 43400 Serdang, Selangor, Malaysia; ^2^Malaysian Research Institute on Ageing (MyAgeing), Universiti Putra Malaysia, 43400 Serdang, Selangor, Malaysia; ^3^Centre for Rehabilitation and Special Needs Studies, Occupational Therapy Programme, Faculty of Health Sciences, Universiti Kebangsaan Malaysia, 50300 Kuala Lumpur, Malaysia

In the article titled “A Systematic Review on Clinimetric Properties of Play Instruments for Occupational Therapy Practice” [1], the authors wish to correct the following sentence in the discussion section of the article:

“Moreover, children struggle to perform pretend play using the given “scrap” materials because the material is foreign to their culture. In addition, the indigenous children also have difficulty to play alone as mostly the play activity happen in pair or group in the indigenous culture.”

The above should be corrected to:

“For the unstructured symbolic play materials, the Elders of the community were consulted in order to modify some of the materials so that the assessment would account for cultural mores and understandings, for example, sand added to the boxes. In addition, in the Australian Aboriginal community where the study was carried out, it was not culturally responsive for children to be assessed one on one, so the children were assessed in pairs.”

## References

[1] Romli M. H., Wan Yunus F. (2020). A Systematic Review on Clinimetric Properties of Play Instruments for Occupational Therapy Practice. *Occupational Therapy International*.

